# Synthesis and characterization of ZnS with controlled amount of S vacancies for photocatalytic H_2_ production under visible light

**DOI:** 10.1038/srep08544

**Published:** 2015-02-25

**Authors:** Gang Wang, Baibiao Huang, Zhujie Li, Zaizhu Lou, Zeyan Wang, Ying Dai, Myung-Hwan Whangbo

**Affiliations:** 1 State Key Laboratory of Crystal Materials, Shandong University, Jinan 250100, People's Republic of China; 2 School of Physics, Shandong University, Jinan 250100, People's Republic of China; 3 Department of Chemistry, North Carolina State University, Raleigh, North Carolina 27695-8205, USA

## Abstract

Controlling amount of intrinsic S vacancies was achieved in ZnS spheres which were synthesized by a hydrothermal method using Zn and S powders in concentrated NaOH solution with NaBH_4_ added as reducing agent. These S vacancies efficiently extend absorption spectra of ZnS to visible region. Their photocatalytic activities for H_2_ production under visible light were evaluated by gas chromatograph, and the midgap states of ZnS introduced by S vacancies were examined by density functional calculations. Our study reveals that the concentration of S vacancies in the ZnS samples can be controlled by varying the amount of the reducing agent NaBH_4_ in the synthesis, and the prepared ZnS samples exhibit photocatalytic activity for H_2_ production under visible-light irradiation without loading noble metal. This photocatalytic activity of ZnS increases steadily with increasing the concentration of S vacancies until the latter reaches an optimum value. Our density functional calculations show that S vacancies generate midgap defect states in ZnS, which lead to visible-light absorption and responded.

In solving the global energy need and environmental pollution, hydrogen has attracted much attention for its potential to replace fossil fuels. Currently, however, pro-duction of hydrogen is mostly based on fossil fuels and on a process requiring high energy consumption^1^,^2^. Since the report of photocatalytic activity of TiO_2_ for hydrogen production, photocatalysis has become a desirable approach toward producing hydrogen with clean, environmentally friendly and economical process. In the past few decades, numerous photocatalysts have been found to exhibit high activities for water splitting^3^,^4^,^5^,^6^. These photocatalysts are mostly active only under UV light, which accounts for only 4% of the total sunlight. For practical applications, therefore, photocatalysts for hydrogen production need to operate under visible light.

ZnS is one of the most widely investigated photocatalysts because it rapidly generates electron-hole pairs under photoexcitation, and exhibits a relatively high activity for H_2_ production under UV light^6^,^7^. ZnS has a hexagonal structure and forms nanosheets or nanorods with large specific surface area^7^,^8^,^9^. Nevertheless, ZnS still has a substantially negative potential for excited electrons, and it does not respond to visible light due to its large band gap (~3.6 eV)^10^. Attempts have been made to extend the optical absorption of ZnS into the visible region by doping it with transition-metal ions (Au, Ni, Cu)^10^,^11^,^12^. However, these composite materials were differently to synthesize and hard to evaluate their intrinsic property of materials. Surface defects can also enhance light harvesting in photocatalytic materials^13^, and can also serve as adsorption sites where a charge transfer to the adsorbed species can prevent the recombination of photogenerated electrons and holes (namely, photogenerated charge carriers). However, when present in excessive amount, defects can act as traps for charge carriers leading to the recombination of photogenerated electrons and holes and hence decreasing the photocatalytic activity^14^,^15^,^16^,^17^. Therefore, controlling the amount of defects is great important to photocatalytic reaction. Vacancies are another kind of intrinsic defects in crystals, which are easily formed in quasi-two-dimensional materials because the exposed atoms on their surface can escape from the lattice hence affecting their physical and chemical properties^18^,^19^. Theoretical investigations have reported that S vacancies can decrease the band gap of ZnS, and the introduction of S vacancies is harder than Zn vacancies in ZnS crystals^20^,^21^. McCloy et al. introduced different vacancies into cubic ZnS crystals by chemical vapor deposition under various atmospheres and studied the change in their lattice parameters^22^. To our knowledge, there has been no study on S vacancies in ZnS with an aim for photocatalytic applications.

In the present work we first controlled amount of S vacancies in ZnS spheres, and characterized the ZnS samples with S vacancies by various experimental techniques and density functional calculations. The ZnS samples with controllable S vacancies were obtained via adding NaBH_4_ as a reducing agent in NaOH solution, and found to possess good photocatalytic activities for H_2_ production without loading noble metal under visible light irradiation. The probable cause for the visible-light photocatalytic activity of these ZnS materials was explored.

## Results

### XRD and SEM characterizations

**Figure 1:**
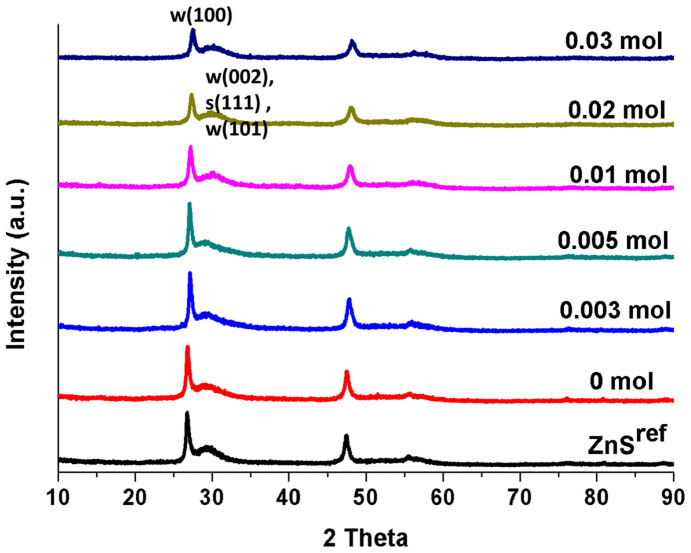
XRD patterns obtained for ZnS^ref^ and the ZnS samples prepared using different amounts of NaBH_4_ (0–0.03 mol), w(100) = wurtzite(100), w(002) = wurtzite (002), s(111) = sphalerite (111), and w(101) = wurtzite (101).

The XRD profiles for the ZnS samples synthesized using NaBH_4_ as reducing agent are given in Fig. 1, which is coordinated with that of ZnS^ref^ reported by Zhang et al^10^. These patterns have a strong and sharp peak at 2θ = 27.2°, which can be indexed as the (100) reflection of wurtzite ZnS, and the broader peak is the overlapping peaks of wurtzite (002), sphalerite (111), and wurtzite (101)^10^,^23^. These suggest that the {001} facet of wurtzite crystals is exposed, which is confirmed by SEM images (see below). The peaks of ZnS decrease with increasing the amount of NaBH_4_ (hence the amount of S vacancies), because S vacancies diminish the crystallinity of ZnS crystals.

**Figure 2:**
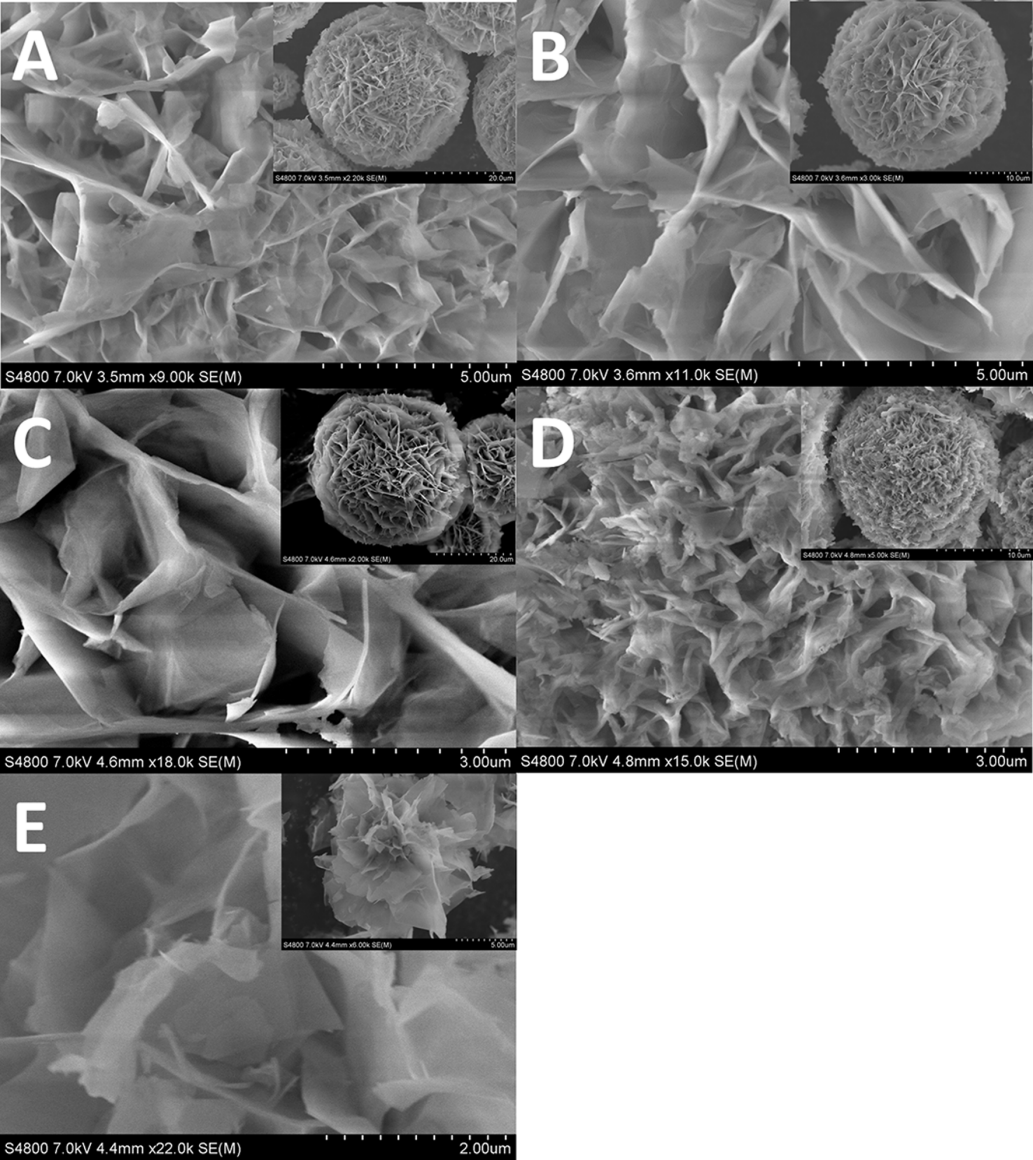
SEM images of the ZnS samples synthesized using different amounts of NaBH_4_. (A) 0.003, (B) 0.005, (C) 0.01, (D) 0.02, and (E) 0.03 mol NaBH_4_.

The morphologies of the prepared ZnS samples are shown in Fig. 2. The nanosheets of microspheres become crimped gradually with increasing the amount of NaBH_4_, because S vacancies enhance the stress in the nanosheets of ZnS. The morphology of the microsphere turns into a blossom as the amount of NaBH_4_ increases to 0.03 mol, in which nanosheets are already unfolded and broken. The morphologies of the microspheres are gradually ruptured, crisp and unfolded as the amount of NaBH_4_ is increased, which may decrease the specific surface areas of the samples reducing the photocatalytic activity.

### Optical absorption

**Figure 3:**
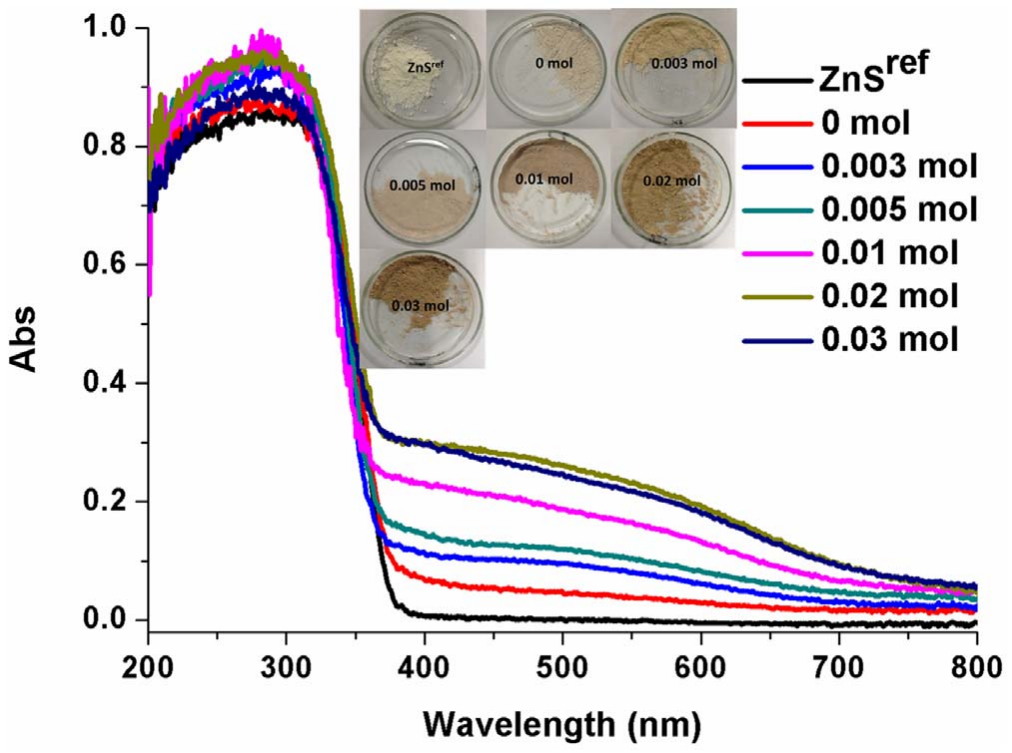
UV-Vis diffuse reflectance spectra of ZnS synthesized using 0–0.03 mol of NaBH_4_.

The UV-Vis diffuse reflectance spectra of the ZnS samples obtained under different conditions are presented in Fig. 3, with their colour changes presented in the inset. It is clearly seen that ZnS^ref^ is a typical direct gap semiconductor with no absorption in the visible light region. The ZnS samples, which obtained by the reaction between Zn and S powder without adding NaBH_4_ (0 mol), exhibited visible light absorption, but one cannot control their visible-light absorption and their photocatalytic activities are low. NaBH_4_ is a strongly reducing agent, and can reduce Zn^2+^ ions thereby creating S vacancies due to the charge balance requirement. Fig. 3 shows the absorption spectra measured for ZnS^ref^ and ZnS prepared without using NaBH_4_ as well as those prepared using NaBH_4_. The amount of NaBH_4_ used for synthesis clearly affects the visible-light absorption; the larger the amount of NaBH_4_, the stronger the absorption in the visible light.

Being a source of H^−^, NaBH_4_ can reduce H_2_O to generate hydrogen. However, under the high-concentration NaOH solution used in our synthesis, the concentration of H^+^ in the solution is negligible so that reduction of H_2_O by H^−^ is negligible. Thus, H^−^ reduces Zn^2+^ ions of the ZnS crystal lattice thereby forming Zn^0^. To keep the charge balance, the amount of S atoms in the ZnS crystal lattice should decrease leading to S vacancies. With increasing the amount of NaBH_4_, more S vacancies are formed resulting in a darker colour of the samples (see the inset of Fig. 3). Therefore, visible-light absorption of ZnS crystals can be tuned by the concentration of S vacancies, which in turn can be controlled by varying the amount of NaBH_4_ in the synthesis process.

### XPS

**Figure 4:**
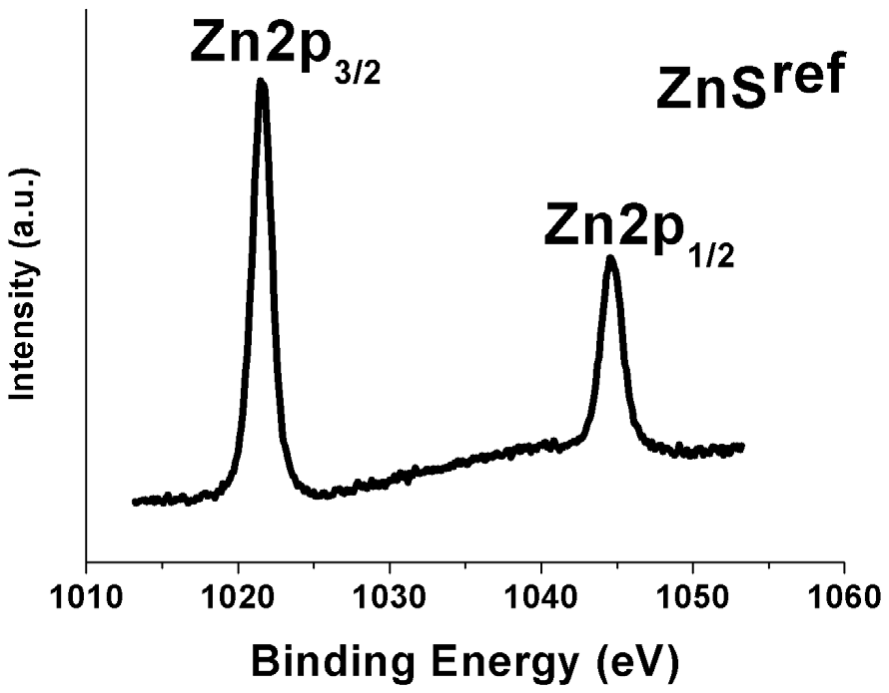
XPS results of ZnS^ref^ sample.

**Figure 5:**
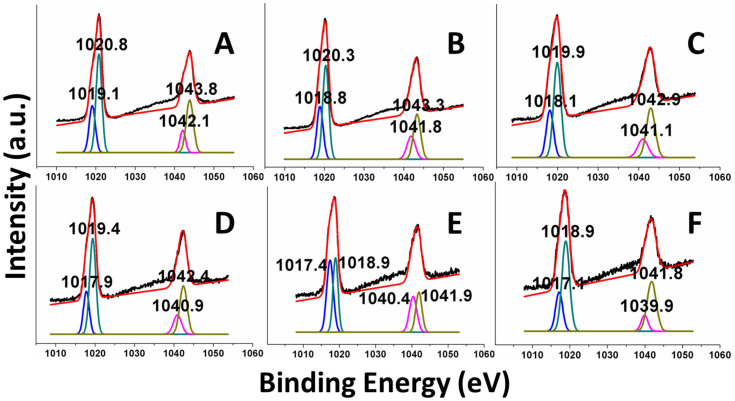
Zn 2p_3/2_ spectra of the ZnS samples synthesized using different amounts of NaBH_4_. (A) 0, (B) 0.003, (C) 0.005, (D) 0.01, (E) 0.02, and (F) 0.03 mol of NaBH_4_.

The chemical states of Zn^2+^ ions in ZnS samples were investigated by XPS (Full XPS survey scan display there was no impurity elements in the sample (Fig. S5, ESI^†^)) As shown in Fig. 4, the binding energies of the Zn 2p_3/2_ and Zn 2p_1/2_ peaks in ZnS^ref^ are about 1021.5 eV and 1044.5 eV, respectively^24^. When Zn and S powders were used for the synthesis without adding NaBH_4_, the resulting ZnS sample weakly absorbs visible light and a lower binding energy for both Zn 2p_3/2_ and Zn 2p_1/2_. This is attributed to the presence of some S vacancies^20^. With increasing the amount of NaBH_4_ during the synthesis, the binding energy for the Zn 2p_3/2_ and Zn 2p_1/2_ peaks of the ZnS samples shift gradually to a lower energy. This indicates that, to a certain degree, the concentration of S vacancies can be controlled by varying the amount of NaBH_4_ used. As depicted in Fig. 5, the Zn 2p_3/2_ peaks can be decomposed into two Gaussian peaks. With increasing the amount of NaBH_4_ from 0 to 0.03 mol, the major Zn 2p_3/2_ peaks change from 1020.8 eV to 1018.9 eV, and the minor Zn 2p_3/2_ peaks from 1019.1 eV to 1017.4 eV. At the same time, the major peaks of Zn 2p_1/2_ change from 1043.8 eV to 1041.8 eV, and the minor Zn 2p_1/2_ from 1042.1 eV to 1039.9 eV. All these peaks are lower in energy than that of ZnS^ref^. It is reasonable to assign the major peaks to the Zn^2+^ ions away from the S vacancy sites, and the minor peaks to those Zn^2+^ ions adjacent to the S vacancy sites, and that the presence of Zn^0^ atoms around the S vacancy sites (see below) lowers the binding energies of the Zn^2+^ ions. EDS results also proved that absence of S element in ZnS samples (Fig S6, ESI^†^). The XPS results of S element in different samples were also investigated, and peaks of S 2p were all shifted to higher energy (Fig S7, ESI^†^).

### Surface areas of ZnS

An important factor affecting the photocatalytic activity of a ZnS sample is its surface area (Table 1). Fig S2 (ESI^†^) shows the typical isotherms of N_2_ adsorption onto the ZnS sample obtained using 0.01 mol of NaBH_4_, with the pore size distributions in the inset. These isotherms exhibit type IV isotherms with hysteresis loops according to the IUPAC classification^25^, which implies the presence of mesopores. The pore size distribution curves (the inset in Fig S2) indicate that the sample possesses mesopores and macropores in a wide size range of 10 to over 90 nm. Use of NaBH_4_ can increase the BET of the sample to a certain degree. The ZnS sample obtained with 0.01 mol of NaBH_4_ possesses a relatively large specific surface area, while use of a greater amount of NaBH_4_ decreases by destroying the microspheres.

### Photocatalytic production of H_2_

**Figure 6:**
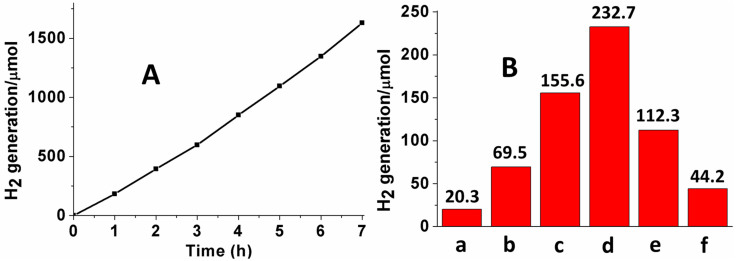
Photocatalytic reactions results. (A) Photocatalytic production of H_2_ by the ZnS sample (0.5 g) obtained using 0.01 mol NaBH_4_ as a function of the visible-light irradiation time. (B) Hydrogen production rate by ZnS samples obtained using different amounts of NaBH_4_ (a = 0 mol, b = 0.003 mol, c = 0.005 mol, d = 0.01 mol, e = 0.02 mol, and f = 0.03 mol).

The ability of the ZnS samples (0.5 g) for photocatalytic hydrogen generations under visible light (λ > 420 nm) irradiation was evaluated by using 100 mL aqueous solution containing Na_2_S (0.25 M) and Na_2_SO_3_ (0.5 M) as sacrificial agents. The H_2_ production of the ZnS sample (obtained with 0.01 mol of NaBH_4_) as a function of the irradiation time is presented in Fig. 6A, which shows that the activity of the sample has no decrease after 7 h irradiation (The XPS results of this sample, before and after photocatalytic reaction, were also show in Fig S4, ESI^†^). The hydrogen production rates of the ZnS samples synthesized using different amounts of NaBH_4_ (0–0.03 mol), displayed in Fig. 6B, show that the production rate increases until the amount of NaBH_4_ reaches 0.01 mol but drops abruptly when it is beyond 0.01 mol. This means that the S vacancies enhance the visible light absorption resulting in an enhanced high photocatalytic activity. However, when present in excess amount, vacancies act as recombination centers for photogenerated carriers decreasing the photocatalytic activity. With increasing the amount of NaBH_4_ to 0.02 and 0.03 mol, lots of hierarchical microspheres are broken, which can also contribute to the precipitous decline of the H_2_ production rate. Cycling photocatalytic experiments of ZnS were also done for evaluating the stability of the ZnS samples (Fig S8, ESI^†^), which exhibited good stability after 3 times photocatalytic reactions.

### PL spectra

**Figure 7:**
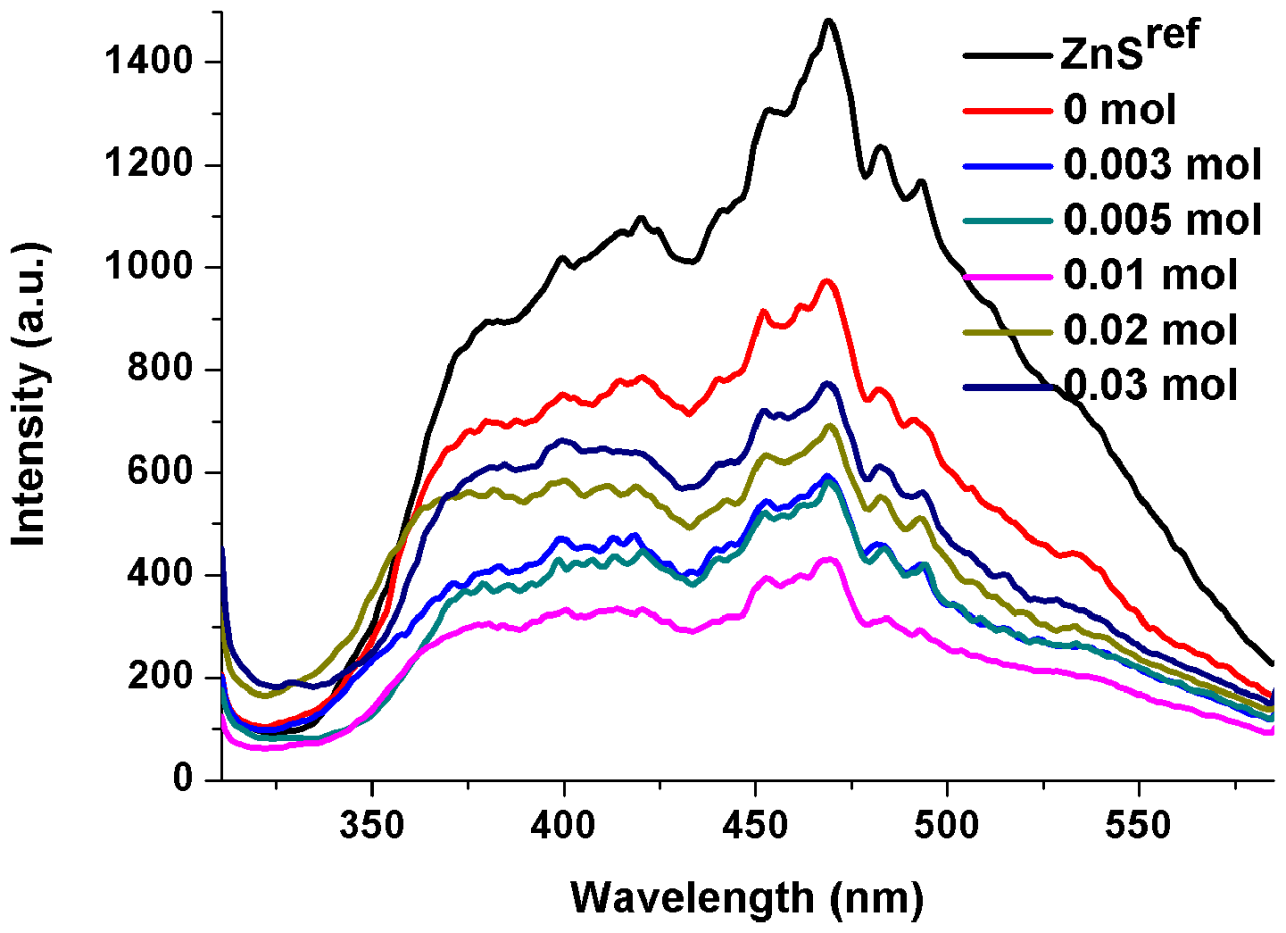
PL spectra of different ZnS samples (λ_exc_ = 300 nm).

The photocatalytic activity is enhanced when photogenerated electron-hole pairs are efficiently separated. PL emission is an effective way of estimating the ability of a sample to separate these photogenerated carriers. The PL spectra of different ZnS samples are presented in Fig. 7, which were carried out on a Hitachi F-4500 fluorescence spectrophotometer at room temperature and obtained with excitation wavelength at 300 nm. It is observed that ZnS^ref^ has the highest intensity of photoluminescence emission, indicating that the photogenerated carriers are quickly recombined. The PL intensity of the ZnS samples decreases with increasing the amount of NaBH_4_ reaches 0.01 mol, but decreases when it is beyond 0.01 mol. It clearly indicates that the S vacancies can become centers for the recombination of photogenerated electrons and holes when present in excessive amount. This finding is consistent with the results of the photocatalytic activities of these ZnS samples.

### Density functional characterization of the defect states

**Figure 8:**
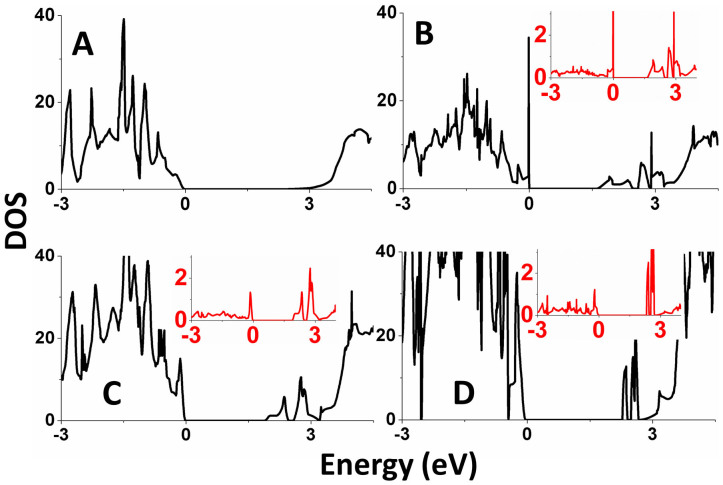
Total DOS and PDOS plots calculated for various ZnS with and without S vacancy. The PDOS plots, shown in the inset, are calculated for the four Zn atoms surrounding the S vacancy. (A) Hexagonal ZnS without S vacancy in which there are 32 atoms per 2 × 2 × 2 supercell. (B) ZnS in which there is one S vacancy per 2 × 2 × 2 supercell. (C) ZnS in which there is one S vacancy per 4 × 2 × 2 supercell. (D) ZnS in which there is one S vacancy per 4 × 4 × 2 supercell.

**Figure 9:**
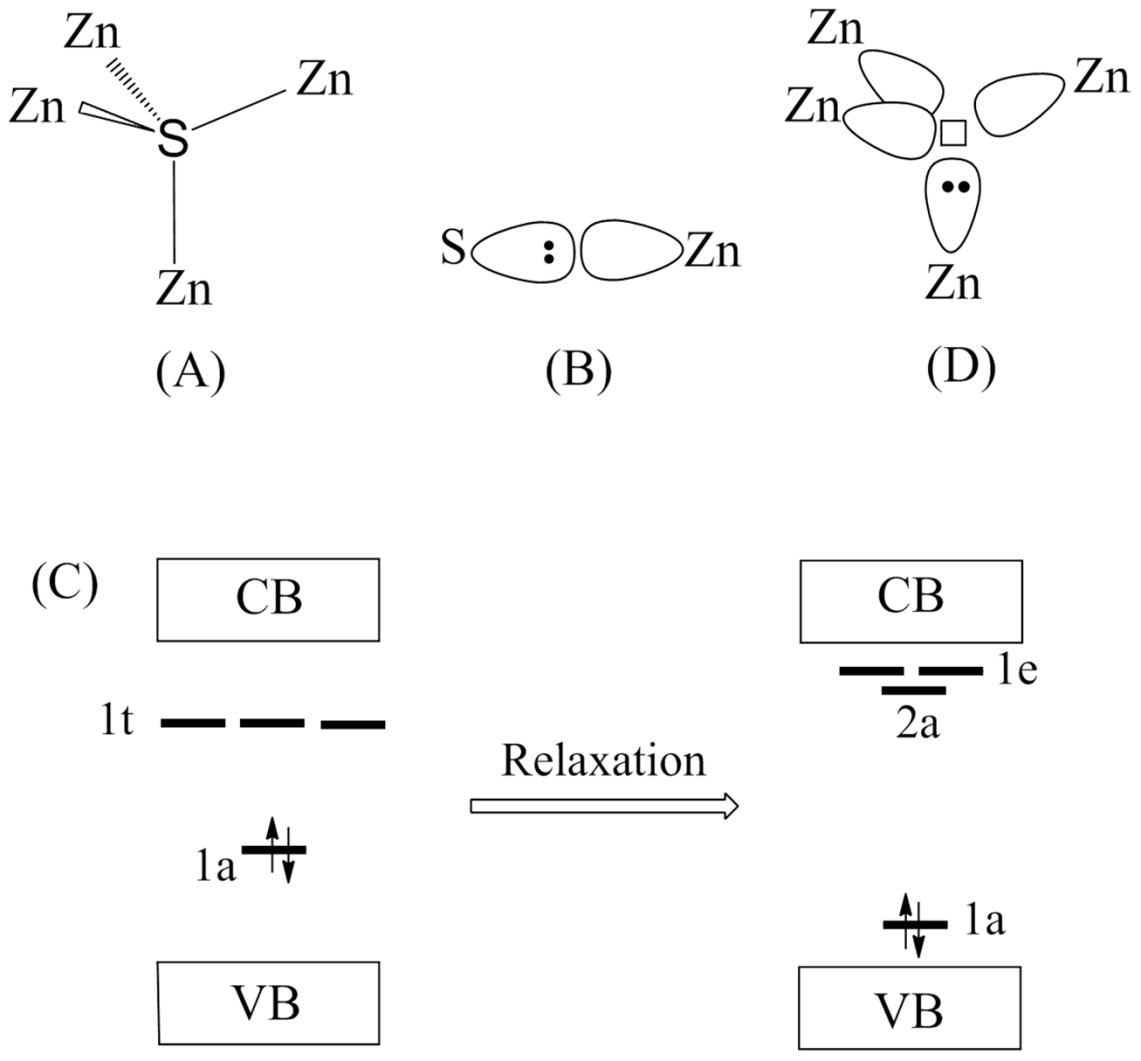
The examination of the relaxed structures. (A) SZn_4_ tetrahedron of ZnS. (B) Zn-S bonding as a combination of a filled sp^3^ hybrid orbital of S^2−^ with an empty sp^3^ orbital of Zn^2+^. (C) Schematic diagram showing the midgap defect states of ZnS created by an S vacancy before (left) and after (right) relaxing the structures around the vacancy site. (D) Overall effect of an S vacancy showing the formation of one of the four ZnS_3_ pyramids surrounding the S vacancy getting two electrons. The VB and CB refer to valence and conduction bands, respectively. Here, for simplicity, it was assumed that the unrelaxed Zn_4_ tetrahedron has a Td symmetry, and the relaxed Zn_4_ tetrahedron a C_3_ symmetry.

The density of states (DOS) plot calculated for ZnS with no S vacancy is presented in Fig. 8A, which shows a band gap of ~3.0 eV. The DOS plots calculated for the model ZnS systems with one S vacancy in the 2 × 2 × 2, 4 × 2 × 2 and 4 × 4 × 2 supercells are presented in Figs. 9B–D, respectively, where the insets show the projected DOS (PDOS) plots calculated for the four Zn atoms surrounding the S vacancy. These plots reveal that the S vacancy generates new states within the band gap of defect-free ZnS. The presence of these midgap defect states causes a visible-light absorption for the ZnS samples with S vacancies. It is of interest to discuss the formation of these defect states from the viewpoint of the local electronic structure around an S vacancy.

The examination of the relaxed structures around the S vacancies show that one of the four ZnS_3_ pyramids surrounding the S vacancy becomes strongly pyramidal (with 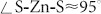) while the remaining three ZnS_3_ pyramids do not undergo a strong change in shape (with 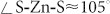). In ZnS each S^2−^ ion is surrounded by four Zn^2+^ ions to form a SZn_4_ tetrahedron (Fig. 9A), and each Zn^2+^ ion surrounded by four S^2−^ ions to form a ZnS_4_ tetrahedron. To a first approximation, the bonding in ZnS can be described in terms of the sp^3^ hybridization for the Zn and S atoms so that each Zn-S bond results from a bonding combination of a filled sp^3^ orbital of S^2−^ (i.e., a sp^3^ lone pair) with an empty sp^3^ orbital of Zn^2+^ (Fig. 9B). When an S atom is removed from a Zn_4_ tetrahedron, each of the four Zn atoms surrounding the S vacancy becomes a ZnS_3_ pyramid with one sp^3^ dangling bond. If the structure around the vacancy site is not relaxed, then the interactions between the four dangling bonds lead to the midgap states 1a and 1t (1a < 1t) of the Zn_4_ tetrahedron lying approximately in the middle of the gap between the valence band maximum (VBM) and conduction band minimum (CBM) (left of Fig. 9C) (see Fig S3, ESI^†^ for the DOS plot in the case of no geometry relaxation around the S vacancy). When the structure around the vacancy site is relaxed, the energy gap between the 1a and 1t levels becomes large with the 1t level splitting (right of Fig. 9C) due to the symmetry lowering. As a consequence, the 1a level comes close to the VBM and becomes doubly filled, while the three empty levels come close to the CBM. In the relaxed structure around an S vacancy, one ZnS_3_ pyramid that becomes strongly pyramidal (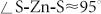) has its dangling bond doubly filled becoming a lone pair (Fig. 9D). Then the optical excitations associated with the filled defect level close the VBM and the empty defect levels below the CBM are responsible for the visible-light absorption of the ZnS samples with S vacancies. Furthermore, trapping of photogenerated electrons and holes by these defect states helps slow down their recombination, thereby enhancing the photocatalytic activities of ZnS samples with S vacancies.

In summary, aimed at finding photocatalytic ZnS systems for H_2_ production operating under visible-light irradiation, we synthesized a new ZnS samples with controlled amount of S vacancies by the hydrothermal reaction of zinc and sulfur powders with NaBH_4_ added as a reducing agent. The concentration of S vacancies in the ZnS samples can be easily controlled by varying the amount of NaBH_4_. The as-prepared ZnS samples exhibit enhanced visible-light absorption with increasing the concentration of S vacancies. The photocatalytic activities of these samples for H_2_ production, evaluated under visible light irradiation, show that the photocatalytic activity increases with increasing the concentration of S vacancies until it reaches the optimum value, but decreases sharply when it goes beyond the optimum value because, when present in excessive amount, S vacancies destroy both crystal structure and morphologies of the samples and also play as recombination sites of photogenerated electrons and holes. Our density functional analysis reveals that S vacancies generate midgap defect states in ZnS, which are responsible for visible-light absorption and also act as trap centers for electrons and holes improving the separation efficiency of photogenerated charge carries. This work provides a novel method to induce and control S vacancies in crystals for enhancing their photocatalytic H_2_ production under visible light.

## Methods

### Matrials

All chemicals in this work (i.e., Zn powder, sublimed sulfur, NaOH, NaBH_4_, Na_2_S·9H_2_O, Na_2_SO_3_) were AR reagents, and were used without any further purification.

### Preparation of ZnS

Samples of ZnS with S vacancies were synthesized by mixing Zn powder and sublimed sulfur in 50 ml of 21 M NaOH solution. After the suspension was cooled to room temperature, a various amount of NaBH_4_ (0–0.03 mol) was added to control the amount of S vacancies in ZnS crystals. After constant stirring for 2 h, the mixed solution was transferred into a 120 ml sealed Teflon-lined autoclave and was heated at 230°C for at least 12 h. The autoclave was slowly cooled down to room temperature, and the obtained sample was washed with distilled water, and was finally dried at 40°C overnight. For the purpose of comparison, we prepared a reference ZnS sample following the method proposed by Zhang et al.^10^ with slight modification^10^, which will be hereafter referred to as ZnS^ref^; a certain amount of ZnO was dissolved in 50 ml of 21 M NaOH solution, and Na_2_S·9H_2_O was added after the solution cooled down. The suspension was transferred into a 120 mL sealed Teflon-lined autoclave and followed by hydrothermal treatment at 230°C for at least 12 h. The sample was also collected after washing and drying.

### Sample characterizations

XRD patterns were recorded on a Bruker AXS D8 Advance powder diffractometer (Cu Kα X-ray tube, λ = 0.154056 nm). The morphologies of the samples were obtained using SEM (Hitachi S-4800). The chemical energy-dispersive X-ray spectrum (EDS) were examined by an energy dispersive X-ray spectrometer equipped in the SEM machine. The surface areas of the as-prepared samples were measured by the BET method using nitrogen adsorption-desorption isotherms on a Micromeritics ASAP 2020 apparatus at liquid nitrogen temperature. UV-Vis diffuse reflectance spectra were obtained for dry-pressed disk samples by using a Shimadzu UV 2550 recording spectrophotometer equipped with an integrating sphere, and BaSO_4_ was used as a reference. PL spectra were carried out on a Hitachi F-4500 fluorescence spectrophotometer at room temperature and the excitation wavelength was 300 nm.

### Details of calculations

Our density functional calculations employed the projector augmented wave method as implemented in the Vienna ab initio simulation package^26^,^27^,^28^,^29^, the generalized gradient approximation of PBE for exchange and correlation corrections^30^,^31^ with plane wave cutoff energies of 400 eV, and a threshold of self-consistent-field energy convergence of 10^−4^ eV. The atomic positions were fully optimized until all the residual forces are smaller than 0.01 eV/Å. The structure of hexagonal ZnS was constructed on the basis of 2 × 2 × 2, 4 × 2 × 2 and 4 × 4 × 2 supercells containing 32, 64 and 128 atoms, respectively. Various model structures of sulfur-deficient ZnS were constructed by removing one sulfur atom from the 32-atom 2 × 2 × 2 supercell (see Fig. S1 ESI^†^), 64-atom 4 × 2 × 2 supercell, and 128-atom 4 × 4 × 2 supercell.

### Photocatalytic activity for H_2_ prodution

A top irradiation vessel connected to a glass-enclosed gas-circulation system was used to evaluate the photocatalytic hydrogen evolution of the ZnS samples. In a typical photocatalytic experiment, 0.5 g sample was suspended in 100 mL aqueous solution containing Na_2_S·9H_2_O (0.35 M) and Na_2_SO_3_ (0.25 M) as sacrificial agents. The temperature was maintained at 5°C. The visible light source was obtained from a 300 W Xe arc lamp (PLS-SXE 300, Beijing Trusttech Co. Ltd.) equipped with an ultraviolet cutoff filter (λ > 420 nm). The amount of H_2_ produced was determined with a gas chromatograph (Varian GC-3800) equipped with thermal conductivity detector.

## Author Contributions

G.W., B.B.H., Z.Z.L. and Z.Y.W. conceived the project, carried out the experiments and analysed the experimental data. Z.J.L., Y.D. and M.-H.W. performed the density functional theory calculations. All authors contributed to writing the manuscript.

## Supplementary Material

Supplementary Information SI

## Figures and Tables

**Table 1:** BET surface areas and pore structures of the ZnS samples obtained using different amounts of NaBH_4_

NaBH_4_ (mol)	Surface area (m^2^/g)	Pore volume (cc/g)	Pore size (Ǻ)
0	113.1	0.55	97.7
0.003	87.4	0.65	148.3
0.005	93.9	0.36	94.2
0.01	129.3	0.61	87
0.02	112.9	0.5	89.1
0.03	90.1	0.53	117.6
