# Supplementary material for: Synthesis and characterization of ZnS with controlled amount of S vacancies for photocatalytic H2 production under visible light
Source: Sci Rep. 2015 Feb 25;5:8544. doi: 10.1038/srep08544 (PMC4339798; doi:10.1038/srep08544)
Supplement: Supplementary Information — SI [file srep08544-s1.pdf]

Supporting information

# Synthesis and characterization of ZnS with controlled amount of S vacancies for photocatalytic $H_2$ production under visible light

Gang Wang,[1] Baibiao Huang,*[1] Zhujie Li,[2] Zaizhu Lou,[1] Zeyan Wang,[1] Ying Dai,[2] and Myung-Hwan Whangbo[3]

[1]State Key Laboratory of Crystal Materials, [2]School of Physics, Shandong University, Jinan 250100, People's Republic of China

[3]Department of Chemistry, North Carolina State University, Raleigh, North Carolina 27695-8205, USA

Correspondence to bbhuang@sdu.edu.cn

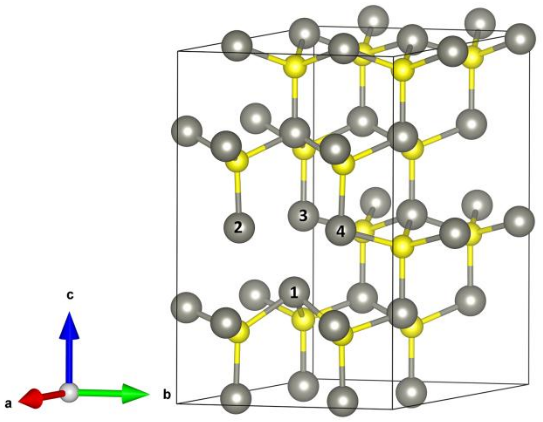


Figure S1. The structure of ZnS with one S atom vacancy in a 32-atom 2×2×2 supercell. The labels 1 – 4 refer to the four Zn atoms surrounding the S vacancy.

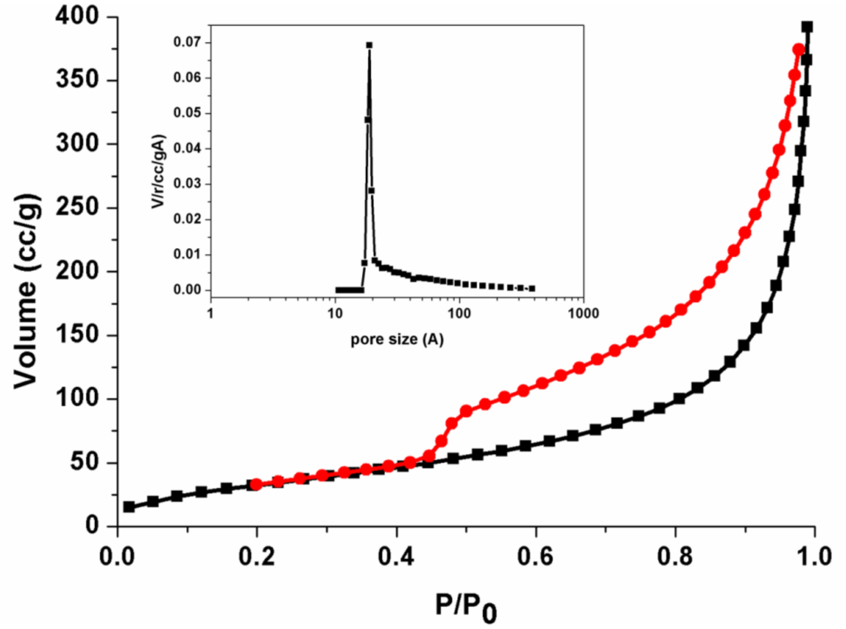


Figure S2. The isotherms of $N_2$ adsorption onto ZnS samples (obtained by using 0.01 mol $NaBH_4$) and together with the pore size distributions (the inset).

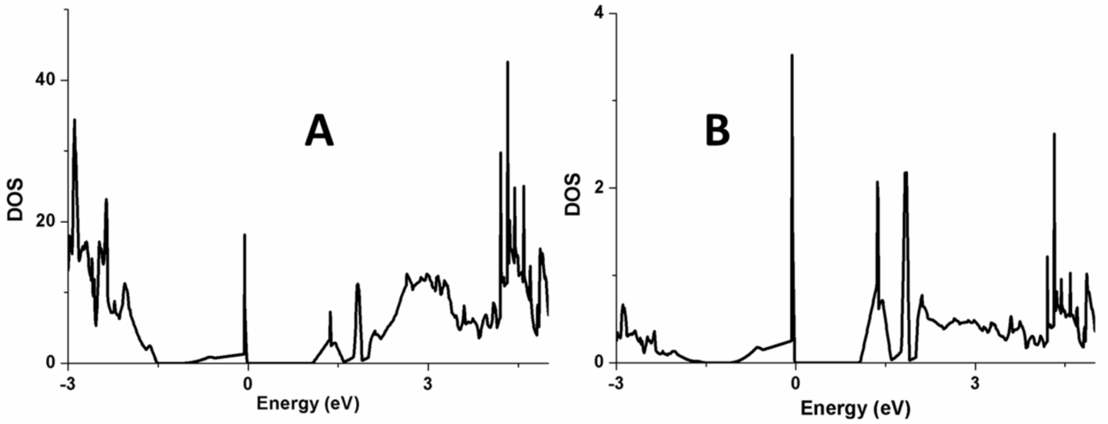


Figure S3. (A) The total DOS plot calculated for ZnS with one S vacancy in a 2×2×2 supercell, for which the geometry relaxation was not included. (B) The projected DOS plot calculated for the four Zn atoms surrounding the S vacancy.

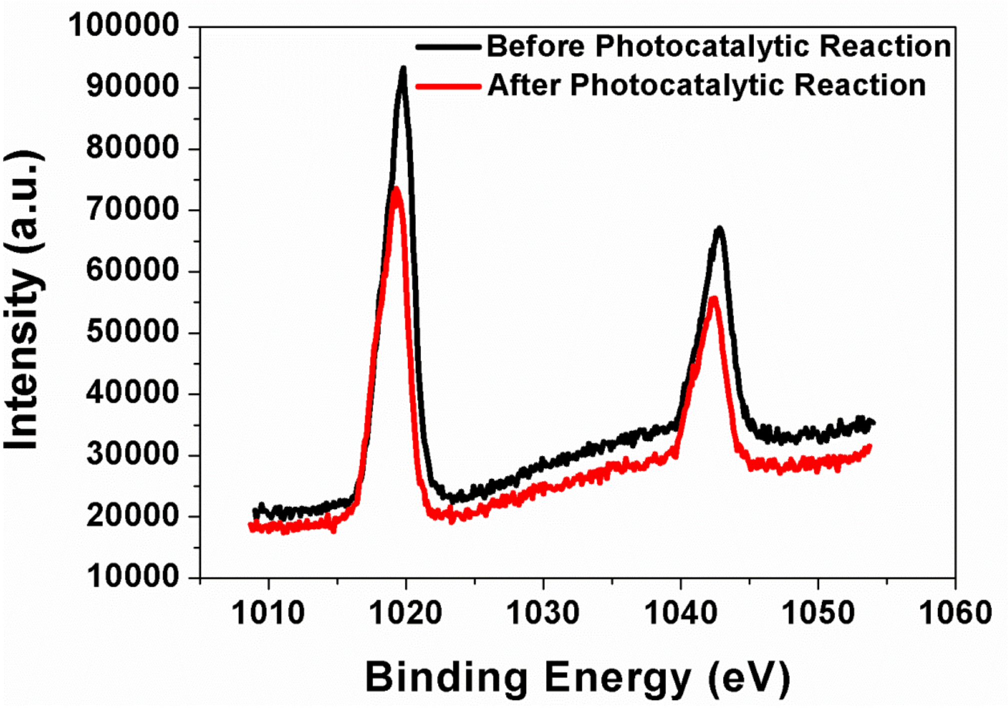


Figure S4. XPS results of ZnS sample (0.01 mol $NaBH_4$) before and after photocatalytic reaction.

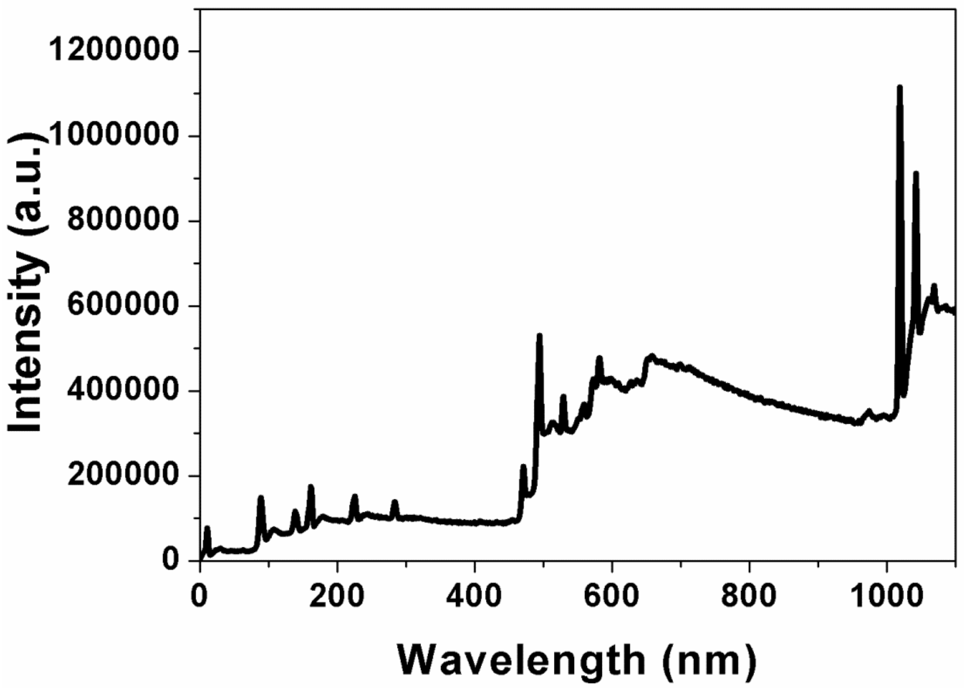


Figure S5. Full XPS survey scan of vacancy ZnS which obtained by adding 0.01 mol $NaBH_4$

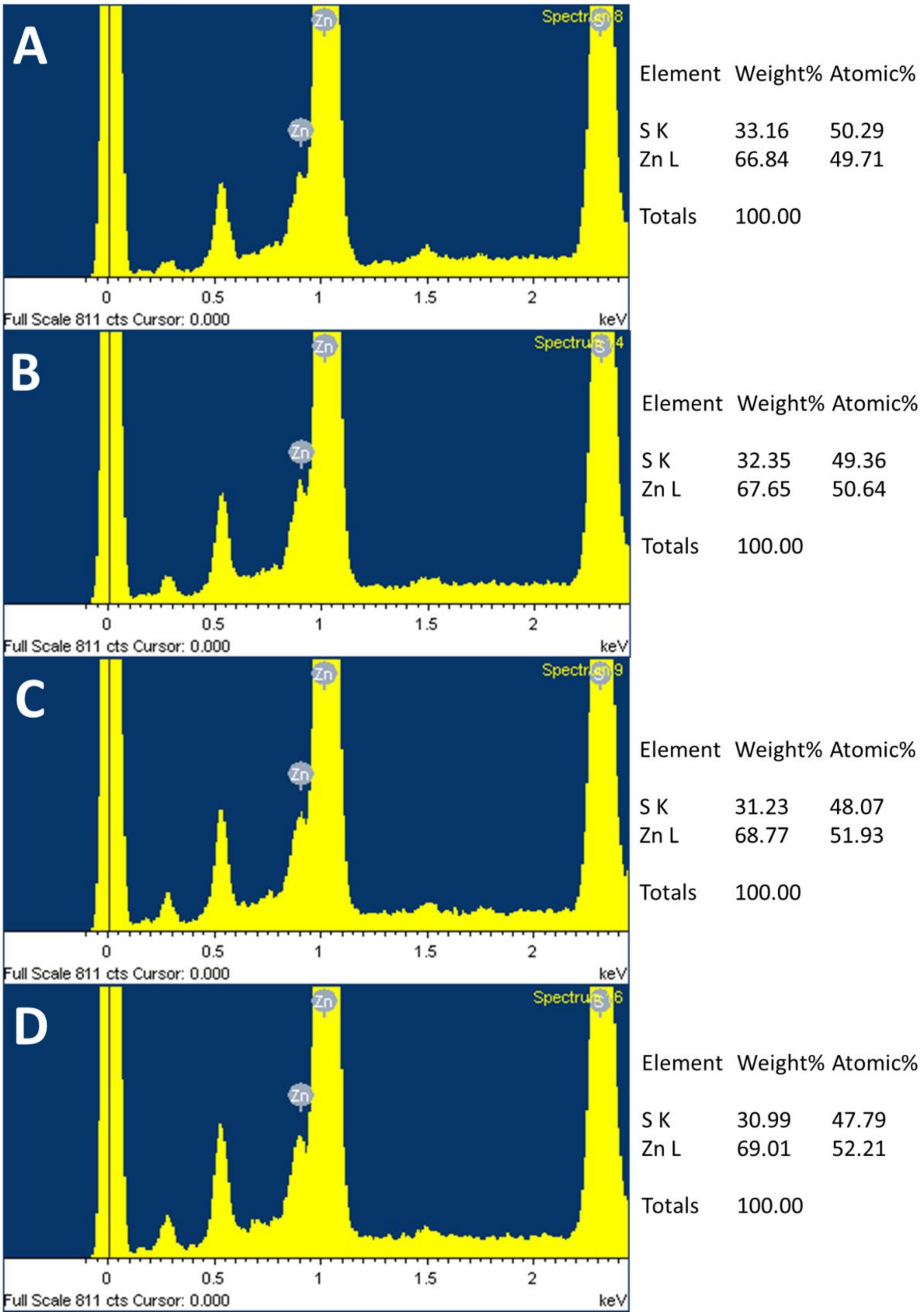


Figure S6. EDS spectrum and detailed information about the composition of different ZnS samples. A is $ZnS^{ref}$, B-D are vacancy ZnS with different $NaBH_4$ added during synthesized (0, 0.01, 0.02 mol).

High resolution XPS of spectrum of S in different ZnS samples are show in Figure S7, the standard binding energy of S $2p_{3/2}$ in $ZnS^{ref}$ was about 161.2 eV [1]. As the increasing of $NaBH_4$, the binding energy of obtained samples gradually shifted to higher direction.

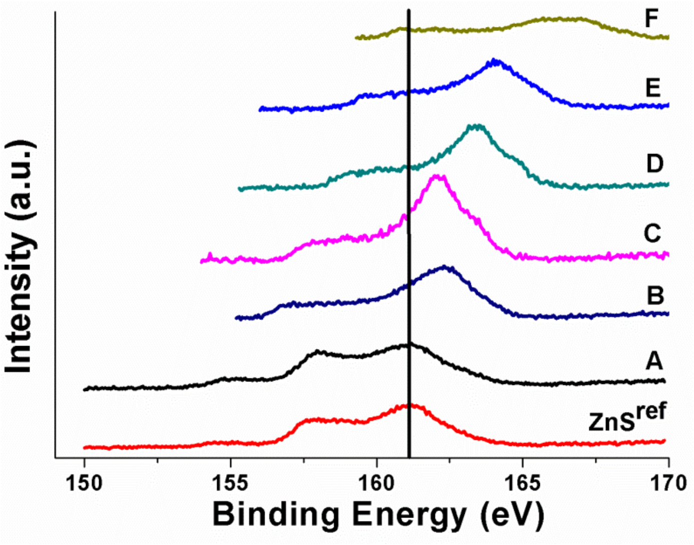


Figure S7. High-resolution XPS spectrum of S in different ZnS samples. (A) 0, (B) 0.003, (C) 0.005, (D) 0.01, (E) 0.02, and (F) 0.03 mol of $NaBH_4$.

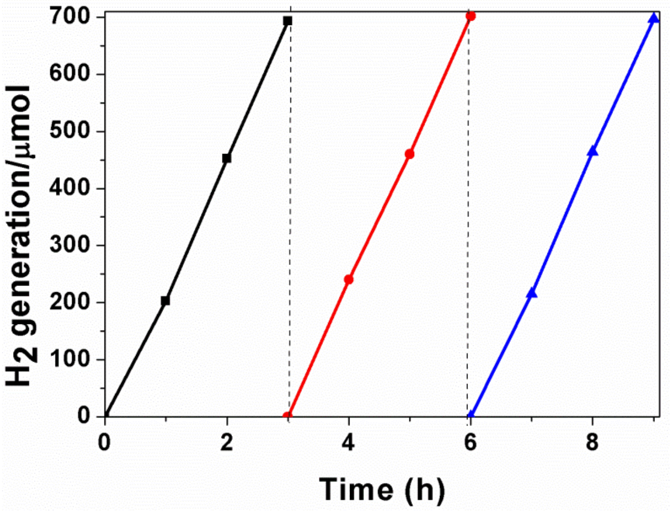


Figure S8. Hydrogen evolution rate of ZnS (0.01 mol) sample in the cyclic test.

[1] Liu, J; Wen, S. M. et. al, Dissolubility and surface properties of a natural sphalerite in aqueous solution. *Miner. Meteall. Proc.* **29**, 113-120 (2012).
